# Effects of intraoperative goal-directed fluid therapy and restrictive fluid therapy combined with enhanced recovery after surgery protocol on complications after thoracoscopic lobectomy in high-risk patients: study protocol for a prospective randomized controlled trial

**DOI:** 10.1186/s13063-020-04983-y

**Published:** 2021-01-07

**Authors:** Zheng Guan, Yanfeng Gao, Qiao Qiao, Qiang Wang, Jingjie Liu

**Affiliations:** 1 Department of Anesthesiology, the First Affiliated Hospital of Xi’an Jiaotong University, Xi’an, Shaanxi People’s Republic of China; 2 Department of Neurology, the Second Affiliated Hospital of Xi’an Jiaotong University, Xi’an, Shaanxi People’s Republic of China

**Keywords:** Thoracoscopic lobectomy, Goal-directed fluid therapy, Enhanced recovery after surgery, Acute kidney injury, Randomized controlled trial

## Abstract

**Background:**

Acute kidney injury (AKI) is a common complication after thoracoscopic lobectomy in high-risk patients due to insufficient intraoperative infusion. Goal-directed fluid therapy (GDFT) is an individualized fluid infusion strategy; the fluid infusion strategy is adjusted according to the patient’s fluid response. GDFT during operation can reduce the incidence of AKI after major surgery. Enhanced recovery after surgery (ERAS) protocol optimizes perioperative interventions to decrease the postoperative complications after surgery. In ERAS protocol of lobectomy, intraoperative restrictive fluid therapy is recommended. In this study, we will compare the effects of intraoperative GDFT with restrictive fluid therapy combined with an ERAS protocol on the incidence of AKI after thoracoscopic lobectomy in high-risk patients.

**Methods/design:**

This is a prospective single-center single-blind randomized controlled trial. Two hundred seventy-six patients scheduled for thoracoscopic lobectomy are randomly allocated to receive either GDFT or restrictive fluid therapy combined with an ERAS protocol at a 1:1 ratio. The primary outcome is the incidence of AKI after operation. The secondary outcomes include (1) the incidence of renal replacement therapy, (2) the length of intensive care unit stay after operation, (3) the length of hospital stay after operation, and (4) the incidence of other complications including infection, acute lung injury, pneumonia, arrhythmia, heart failure, myocardial injury after noncardiac surgery, and cardiac infarction.

**Discussion:**

This is the first study to compare intraoperative GDFT with restrictive fluid therapy combined with an ERAS protocol on the incidence of AKI after thoracoscopic lobectomy in high-risk patients. The hypothesis is that the restrictive fluid therapy is noninferior to GDFT in reducing the incidence of AKI, but restrictive fluid therapy is simpler to apply than GDFT.

**Trial registration:**

ClinicalTrials.govNCT04302467. Registered on 26 February 2020

**Supplementary Information:**

The online version contains supplementary material available at 10.1186/s13063-020-04983-y.

## Background

The term acute kidney injury (AKI) is used to describe a rapid deterioration (hours to days) of renal function. AKI is not an uncommon disorder and is associated with considerable morbidity and mortality [1]. Lobectomy is the primary treatment of lung cancer, and the morbidity of acute kidney injury (AKI) after lobectomy was about 5.9% [2]. AKI increased the postoperative mechanical ventilation time, the length of hospital stay, and the mortality within 30 days after operation [3]. So it is important to reduce the incidence of AKI after lobectomy.

Fluid management is important during operation; there are too many complications of excessive intraoperative infusion that it has been abandoned by anesthesiologists [4]. Intraoperative restrictive infusion can induce organ hypoperfusion, even lead to organ dysfunction and failure, such as AKI. A study showed that restrictive infusion during major abdominal surgery increased the incidence of renal replacement therapy [5]. Another study of 92,094 noncardiac surgery patients also found that intraoperative restrictive infusion was related to the occurrence of postoperative AKI [6].

Goal-directed fluid therapy (GDFT) is an individualized fluid infusion strategy; the fluid infusion strategy is adjusted according to the patient’s fluid response. The patient’s fluid response can be judged by stroke volume variation (SVV) or cardiac index (CI). Meta-analysis showed that GDFT could reduce the incidence of postoperative AKI [7]. GDFT could also reduce the incidence of postoperative AKI after major abdominal surgery [8]. So the American Society for Enhanced Recovery recommends to adopt GDFT during operation in critical surgical patients [9].

Enhanced recovery after surgery (ERAS) optimizes perioperative interventions to decrease postoperative complications and to facilitate postoperative recovery. In 2019, the Enhanced Recovery After Surgery Society and European Society of Thoracic Surgeons recommend restrictive fluid therapy during lobectomy as part of an ERAS protocol in lung surgery [10]. A retrospective study showed there was no relationship between restrictive fluid therapy and AKI after thoracic surgery [11]. But there is no prospective study of restrictive fluid therapy on the incidence of AKI after thoracic surgery in high-risk patients.

This prospective single-center single-blind randomized controlled trial will compare intraoperative GDFT with restrictive fluid therapy combined with an ERAS protocol on the incidence of AKI after thoracoscopic lobectomy in high-risk patients. The secondary outcomes of this study include the incidence of renal replacement therapy, length of intensive care unit (ICU) stay after operation, length of hospital stay after operation, and incidence of other complications including infection, acute lung injury (ALI), pneumonia, arrhythmia, heart failure, myocardial injury after noncardiac surgery (MINS), and cardiac infarction.

## Methods/design

This is a prospective single-center single-blind randomized controlled trial; the protocol was approved by the Ethics Committee of the First Affiliated Hospital of Xi’an Jiaotong University (XJTU1AF2019LSL-012) and was registered in ClinicalTrials.gov (NCT04302467) before the first patient will be enrolled. This trial will be conducted in a tertiary teaching hospital (the First Affiliated Hospital of Xi’an Jiaotong University) in Shaanxi, PR China. The protocol conforms to the Standard Protocol Items: Recommendations for Interventional Trials (SPIRIT) guidelines (Table 1 and SPIRIT checklist). The schedule of enrollment, intervention, and assessment is based on the SPIRIT diagram.

**Table 1:**
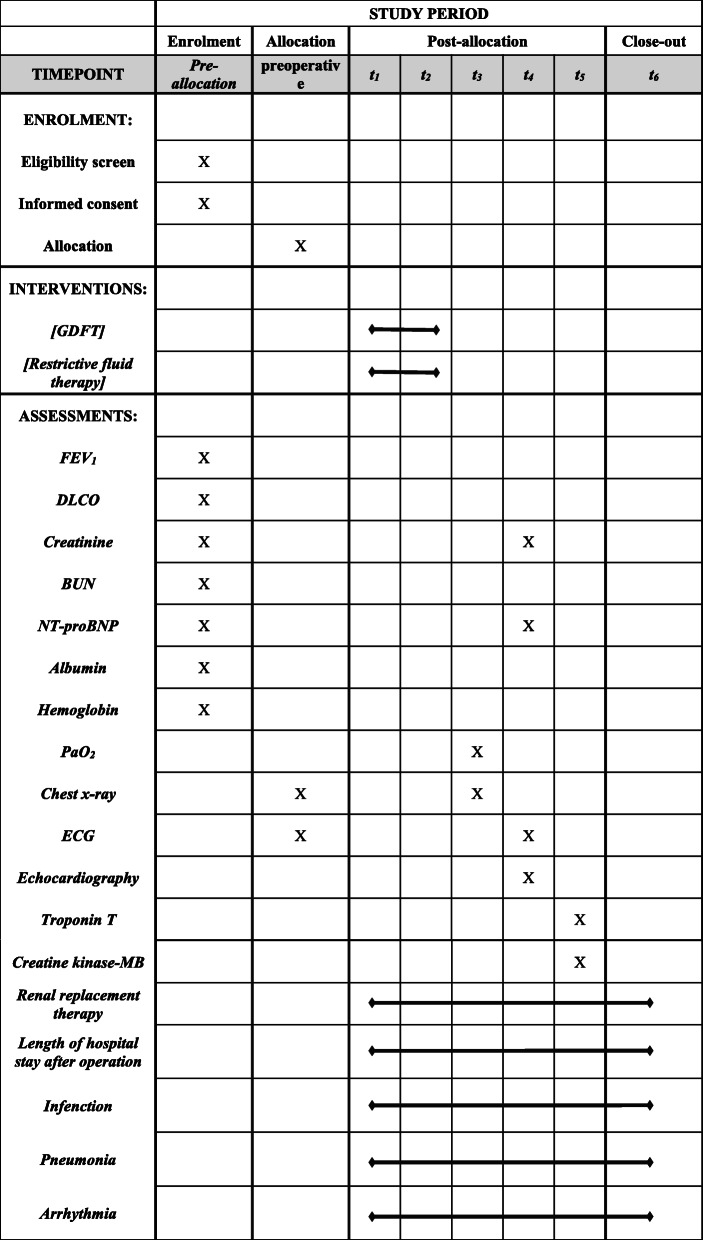
The SPIRIT flow diagram: the schedule of enrollment, interventions, and assessments. *t*_1_ is the beginning of anesthesia. *t*_2_ is the ending of anesthesia. *t*_3_ is 24 h after operation. *t*_4_ is 48 h after operation. *t*_5_ is 7 days after operation. *t*_6_ is discharge or 30 days after operation, depending on which one occurs first

### Hypotheses of the study

This is a noninferiority study, and the hypothesis is that there is noninferiority of restrictive fluid therapy combined with an ERAS protocol than GDFT combined with an ERAS protocol in reducing the incidence of AKI after thoracoscopic lobectomy in high-risk patients.

### Participants

Patients aged more than 18 years old who are scheduled for thoracoscopic lobectomy are eligible for enrollment to this trial. Candidate participants who fulfill the inclusion criteria will be recruited after hospitalization. The following are the inclusion and exclusion criteria:

Inclusion criteria: patients who sign the informed consent of the trial voluntarily and comply any of the following inclusion criteria will be asked for inclusion:

- Age > 70 years old
- Forced expiratory volume in 1 s (FEV_1_) < 60%
- Carbon monoxide lung diffusion capacity (DLCO) < 60%
- History of coronary artery disease

Exclusion criteria: patients who comply any of the following criteria may not be enrolled:

- Patients refused to participate
- Combined with kidney dysfunction: creatinine > 176 μmol/L and/or blood urea nitrogen (BUN) > 7.1 mmol/L
- Combined with cardiac dysfunction: NT-proBNP > 300 ng/L
- Combined with systemic or local infection
- Severe dehydration, malnutrition: albumin < 30 g/L and/or hemoglobin < 100 g/L
- Allergy to hydroxyethyl starch

### Ethics, consent, and permissions

Trained research assistants will screen patients who are scheduled for elective thoracoscopic lobectomy. Participants’ or their representatives’ written informed consents will be obtained by anesthesiologists before they are enrolled in the study. The informed consent form and information materials are available from the corresponding author on request.

### Randomization and blinding

**Fig. 1:**
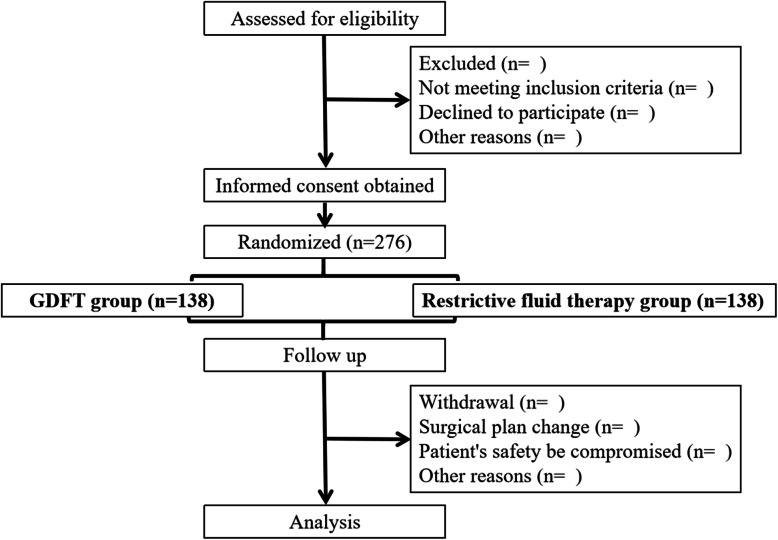
Experiment flow chart

Participants will be randomly allocated to one of the two groups: (1) the GDFT group and (2) the restrictive fluid therapy group. They will be allocated at a 1:1 ratio using random numbers generated by Microsoft Excel (Fig. 1).

The specific method of random grouping is as follows: enter a set of data from 1 to 276 in column A, enter the formula “=RAND()” in column B1, and drop down formula from B1 to B276 to generate random data, arranging the data in column B in ascending order. Patients will be numbered serially according to the order of admission, the number above the line A138 (include A138) will be divided into the GDFT group, and the number below the line A138 will be divided into the restrictive fluid therapy group.

The randomization will be performed by an assistant who is not involved in this study. Allocation details will be kept in sealed envelopes marked by serial number and will be blinded to all participants but the anesthesiologists. Before the induction of anesthesia, the envelopes will be opened by an anesthesiologist. The data assessment and analysis will be performed by an independent research staff supervised by an independent statistician. The patients, the clinical researchers for the collection of data, and the postoperative follow-up team will all be blinded to group allocation.

The serious adverse events include anaphylactic shock, cardiac arrest, and event that causes disability. If serious adverse events occur, interventions should be interrupted if necessary and the patient should be followed up, the blinding will be opened, the events will be reported to the institutional review board, and compensation will be performed to those who suffer harm from trial participation. Medical data will still be collected and analyzed. If general adverse events occur, the patients would not need to be unblinded.

### Withdrawal, dropout, and discontinuation

We will make best effort to maintain the participants’ interest throughout the perioperative period. Participants will always stay in the trial unless he/she withdraws participation by himself/herself. The interventions should be interrupted if the participants’ safety will be compromised or the surgical plan changes unexpectedly. The researchers have no right to decide the withdrawal of the participants. The withdrawal participants will also be followed up, and the data that was collected prior to patient withdrawal could be used for analysis.

### Confidentiality

Participants’ private information will not be collected. Only the study code will be collected. The data collected will be kept confidential until they are required for analysis. The data collected will be stored under encryption for 2 years after the completion of the study.

### Interventions

All participants will perform respiratory functional exercise, be encouraged to quit smoking (the patients who do not quit smoking will still complete the study), and improve nutritional status before the operation. They will fast solid food for 6 h and fast water for 2 h before operation. General anesthesia will be inducted with sufentanil, propofol, and rocuronium without premedication, and it will be maintained with continuous infusion of propofol and remifentanil and intermittent injection of rocuronium. One-lung ventilation will be employed using a double-lumen tube during operation. A lung-protective ventilation strategy will be used. The tidal volume will be 4–6 mL/kg ideal body weight, and the positive end-expiratory pressure will be adjusted according to individual respiratory mechanics and is usually in the range of 5–10 cmH_2_O. The recruitment maneuver of 25 cmH_2_O for 30 s will be performed before two-lung ventilation. After general anesthesia induction, paravertebral nerve block will be performed under ultrasound guidance. Patient-controlled analgesia and oral analgesics will be used for postoperative analgesia. Patients will resume drinking and eating as early as possible, and they also will get out of bed as soon as possible.

**Fig. 2:**
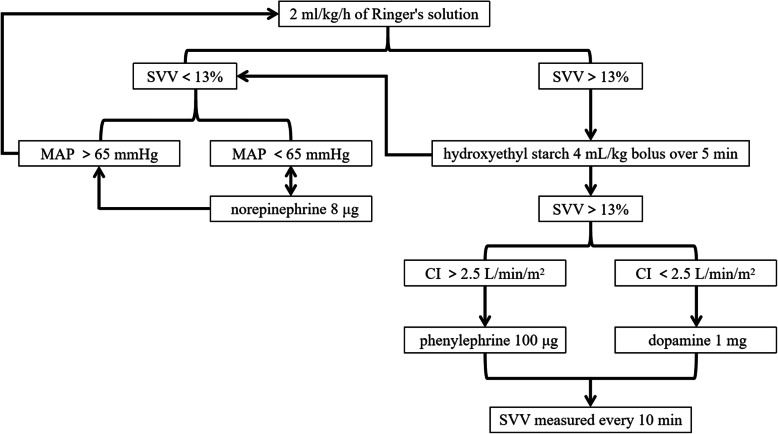
Fluid therapy process in the GDFT group

In the GDFT group, the radial arterial catheter will be connected to FloTrac/Vigileo sensor (Edwards Lifesciences, Irvine, CA, USA). The SVV and CI will be monitored continuously. Fluid maintenance with 2 mL/kg/h of Ringer’s solution of sodium acetate, when SVV > 13%, and 4 mL/kg bolus of hydroxyethyl starch will be infused within 5 min. If SVV falls below 13%, the bolus will be suspended. If SVV is still more than 13%, 100 μg of phenylephrine will be administered when CI is more than 2.5 L/min/m^2^, or 1 mg of dopamine will be administered when CI is less than 2.5 L/min/m^2^. When SVV < 13%, but mean arterial pressure (MAP) < 65 mmHg, 8 μg of norepinephrine will be administered. The hemodynamic status will be repeatedly measured every 10 min (Fig. 2).

In the restrictive fluid therapy group, fluid maintenance with 2 mL/kg/h of Ringer’s solution of sodium acetate and hydroxyethyl starch will be infused to supply blood loss, and the ratio of hydroxyethyl starch to blood loss is 1:1 (mL). Norepinephrine will be administered at a rate of 0.01–0.1 μg/kg/min to maintain MAP > 65 mmHg. The infusion rate will be adjusted by anesthesiologists according to their experience.

We will do our best to improve adherence to intervention protocols, including training of anesthesiologists, operating room nurses, and specialist nurses. Treatment checklist will be used to ensure every intervention will be implemented accurately.

### Measurements

Assistants who are not otherwise involved in the study will assess the outcomes. The primary outcome is the incidence of AKI 48 h after operation. The secondary outcomes include:

1. Incidence of renal replacement therapy 30 days after operation
2. Length of ICU stay after operation
3. Length of hospital stay after operation
4. Incidence of other complications including ALI 24 h after operation, heart failure 48 h after operation, MINS and cardiac infarction 7 days after operation, infection, pneumonia, and arrhythmia 30 days after operation

#### The definition of primary and secondary outcomes

AKI is defined as 50% relative increase or absolute increase 0.3 mg/dL (26.5 μmol/L) of creatinine from the baseline during the first two postoperative days.

ALI is defined as oxygen partial pressure/fraction of inhaled oxygen (PaO_2_/FiO_2_) < 300 mmHg 24 h after operation, combined with bilateral lung infiltration, but cardiogenic pulmonary edema should be excluded.

Pneumonia is defined as new-onset, progressive, or persistent infiltration, consolidation, and cavitation in the chest x-ray. Combining at least one of the following features: (1) temperature > 38 °C without other recognized causes, (2) white blood cell count < 4 × 10^9^/L or > 12 × 10^9^/L, and (3) altered mental status without other recognized causes if age more than 70 years old. And combining at least two of the following features: (1) new onset of purulent sputum, sputum character changed, respiratory secretion increased, or endotracheal suction requirement increased; (2) new-onset or progressive cough, dyspnea, or tachypnea; (3) rales or bronchial breathing sounds appeared; and (4) gas exchange worsened.

Heart failure is defined as NT-proBNP is more than 450 ng/L if age is less than 50 years old or NT-proBNP is more than 900 ng/L if age is more than 50 years old.

MINS is defined as troponin T is more than 0.03 ng/mL and/or creatine kinase-MB is more than 8.8 ng/mL within the 7 days after operation.

Cardiac infarction is defined as troponin T elevated, combining at least one of the ischemic symptoms: (1) new or presumed new Q waves, (2) morphological changes of ST segment or T wave, (3) new-onset left bundle branch block, and (4) new or presumed new regional wall motion abnormality.

### Sample size

The primary outcome of this trial is the incidence of AKI after operation. Another study showed that the incidence of AKI was 4% and 2.9% in liberal infusion and GDFT, respectively, and the efficacy of the intervention was 27.5% [12]. The hypothesis is that restrictive fluid therapy combined with an ERAS protocol is noninferior to GDFT in reducing the incidence of AKI. The sample size for this study was calculated to achieve a statistical power of 0.8 and alpha error of 0.05 using a two-sided test. Considering a dropout rate of 10%, 138 patients are required in each group. PASS (version 15.0.3, NCSS, LLC, Kaysville, UT, USA) was used to estimate the sample size.

### Data collection and management

The data will be collected using the case report form. All the data will be deposited safely in the in-built server in the First Affiliated Hospital of Xi’an Jiaotong University with full confidentiality.

All the data will be monitored by the data monitoring committee (DMC) of the First Affiliated Hospital of Xi’an Jiaotong University. The DMC is composed of the staffs in the clinical research center, and they will inspect the data in regular follow-up period. The DMC is independent from the sponsor, and they have no competing interests. The charter of DMC can be obtained from the clinical research center of the First Affiliated Hospital of Xi’an Jiaotong University if necessary.

### Statistical analysis

Normally distributed data will be presented as the mean ± standard deviation. Categorical data will be presented as the number and the percentage.

The primary outcome (the incidence of AKI after operation) will be compared between the two groups using Fisher’s exact test due to the low incidence rate. The secondary outcomes will be compared between the two groups using independent samples Student’s *t* tests for normally distributed continuous data and using Pearson’s chi-square test for categorical data. All statistical tests are two-sided. *P* value < 0.05 will be considered as there is a statistical difference. The statistical analysis will be performed using SPSS Statistics software by a statistician who is not involved in the study.

## Discussion

AKI is a severe complication, associated with a high incidence of morbidity and mortality. Over the years, many definitions of decreased kidney function have been published; in this study, we used the Kidney Disease Improving Global Outcomes (KDIGO) guideline for the identification of AKI [13]; this definition can increase physicians’ attention to kidney dysfunction as early as possible. In this study, we focus on high-risk patients, because elderly, low FEV_1_, and coronary artery disease all are risk factors of AKI after thoracic surgery [2], and cardiopulmonary complications are also common after thoracoscopic lobectomy in high-risk patients [14].

Fluid management is important during operation. Ideal intraoperative fluid infusion has been identified as a main factor to avoid the postoperative complications. The MAP, central venous pressure, and urine output are slightly related to the hemodynamic goals of fluid management [15]. GDFT is based on the measurement of functional hemodynamic variables, and it can regulate fluid management precisely. GDFT can decrease the incidence of postoperative complications in different kinds of operation [16]. In this study, we used SVV and CI to monitor both the patient’s fluid response and cardiac function, and fluid response can be judged by the variety of SVV during bolus infusion. When there is no fluid response, vasoactive drugs will be used to maintain cardiac function and blood pressure normal. SVV and CI can be obtained by arterial contour analysis using FloTrac/Vigileo sensor (Edwards Lifesciences, Irvine, CA, USA); only experienced anesthesiologists can accurately interpret parameters and make the right intervention, so there may be some bias between different anesthesiologists.

Restrictive fluid therapy is applied in thoracic surgery to reduce the incidence of acute lung injury. But restrictive fluid therapy during operation can induce hypovolemia, hypotension, and organ hypoperfusion, and kidney hypoperfusion is one cause of AKI. The ERAS protocol in lung surgery shortens the fast time before operation, so the dehydration before operation can be avoided. In our study, norepinephrine will be administered to maintain MAP > 65 mmHg, vasopressor is helpful to counteract vasodilation, and the relative hypovolemia caused by anesthetics can be reversed [17]. So restrictive fluid therapy combined with an ERAS protocol is safe and feasible during lobectomy, and it is simpler to apply than GDFT.

This is the first prospective randomized controlled trial to compare intraoperative GDFT with restrictive fluid therapy combined with an ERAS protocol on the incidence of AKI after thoracoscopic lobectomy in high-risk patients. It is expected that the results of this study will provide a simpler fluid therapy strategy during lobectomy.

### Trial status

Following the approval of the study protocol (XJTU1AF2019LSL-012), the current protocol version was approved on 9 December 2019, and the recruitment of subjects will be commenced in April 2020 and will be completed in March 2022.

## Supplementary Information

**Additional file 1.** SPIRIT 2013 Checklist: Recommended items to address in a clinical trial protocol and related documents.

## Data Availability

The datasets analyzed during the current study will be available from the corresponding authors on reasonable request.

## Abbreviations

AKI: Acute kidney injury
GDFT: Goal-directed fluid therapy
SVV: Stroke volume variation
CI: Cardiac index
ERAS: Enhanced recovery after surgery
ICU: Intensive care unit
ALI: Acute lung injury
MINS: Myocardial injury after noncardiac surgery
FEV_1_: Forced expiratory volume in 1 s
DLCO: Carbon monoxide lung diffusion capacity
BUN: Blood urea nitrogen
ECG: Electrocardiography
MAP: Mean arterial pressure
PaO_2_: Oxygen partial pressure
FiO_2_: Fraction of inhaled oxygen
